# Methods to discriminate primary from secondary dengue during acute symptomatic infection

**DOI:** 10.1186/s12879-018-3274-7

**Published:** 2018-08-07

**Authors:** Thi Hanh Tien Nguyen, Hannah E. Clapham, Khanh Lam Phung, Thanh Kieu Nguyen, The Trung DInh, Than Ha Quyen Nguyen, Van Ngoc Tran, Stephen Whitehead, Cameron Simmons, Marcel Wolbers, Bridget Wills

**Affiliations:** 1 Hospital for Tropical Diseases, Oxford University Clinical Research Unit, Ho Chi Minh City, Vietnam; 2 Centre for Tropical Medicine, Nuffield Department of Medicine, University of Oxford, Oxford, UK; 3 Nossal Institute of Global Health, School of Population and Global Health, University of Melbourne, Parkville, VIC Australia; 4 Hospital for Tropical Diseases, Ho Chi Minh City, Viet Nam; 5 Laboratory of Infectious Diseases, National Institutes of Allergy and Infectious Diseases, Bethesda, MD USA

**Keywords:** Dengue, Immune status, Algorithms, ELISA, IgM, IgG

## Abstract

**Background:**

Dengue virus infection results in a broad spectrum of clinical outcomes, ranging from asymptomatic infection through to severe dengue. Although prior infection with another viral serotype, i.e. secondary dengue, is known to be an important factor influencing disease severity, current methods to determine primary versus secondary immune status during the acute illness do not consider the rapidly evolving immune response, and their accuracy has rarely been evaluated against an independent gold standard.

**Methods:**

Two hundred and ninety-three confirmed dengue patients were classified as experiencing primary, secondary or indeterminate infections using plaque reduction neutralisation tests performed 6 months after resolution of the acute illness. We developed and validated regression models to differentiate primary from secondary dengue on multiple acute illness days, using Panbio Indirect IgG and in-house capture IgG and IgM ELISA measurements performed on over 1000 serial samples obtained during acute illness.

**Results:**

Cut-offs derived for the various parameters demonstrated progressive change (positively or negatively) by day of illness. Using these time varying cut-offs it was possible to determine whether an infection was primary or secondary on single specimens, with acceptable performance. The model using Panbio Indirect IgG responses and including an interaction with illness day showed the best performance throughout, although with some decline in performance later in infection. Models based on in-house capture IgG levels, and the IgM/IgG ratio, also performed well, though conversely performance improved later in infection.

**Conclusions:**

For all assays, the best fitting models estimated a different cut-off value for different days of illness, confirming how rapidly the immune response changes during acute dengue. The optimal choice of assay will vary depending on circumstance. Although the Panbio Indirect IgG model performs best early on, the IgM/IgG capture ratio may be preferred later in the illness course.

**Electronic supplementary material:**

The online version of this article (10.1186/s12879-018-3274-7) contains supplementary material, which is available to authorized users.

## Background

Dengue is the most widely distributed mosquito-borne human viral disease and represents a major public health burden globally. An estimated 390 million infections occur each year, of which around 100 million are symptomatic [1]. Although the majority of symptomatic individuals recover after a short illness, a small proportion of patients develop severe complications that can be life threatening. There are four dengue viral serotypes (DENV1–4), all of which may cause severe disease. Although the pathogenesis of severe dengue remains incompletely understood, it is clear that following an initial (primary) infection with one viral serotype, a subsequent infection with a different serotype (secondary infection) is more likely to result in severe disease [2, 3]. A number of virological and immunological parameters that are thought to contribute to dengue pathogenesis differ between individuals with primary and secondary infections [4–7], and differentiating between primary and secondary dengue is important for pathogenesis and epidemiological research. However, it also has potential utility in clinical practice, especially early in the disease evolution when knowledge of the immune status of a confirmed dengue case could help clinicians decide on the need for hospitalisation or frequency of follow-up, and might improve the performance of risk prediction algorithms for severe disease.

Several serological methods have been developed to categorise dengue infections as either primary or secondary. Although Haemagglutination Inhibition Assays (HI assays) have been traditionally considered the gold standard, the technique is complicated and time-consuming to perform, and requires experienced technical staff and samples collected in late convalescence. By comparison, serological testing using ELISA (enzyme-linked immunosorbent assay) techniques to measure IgM and/or IgG levels is technically much simpler, and a number of algorithms now use ELISA titres measured on a single specimen to define immune status. However, the range of techniques and definitions used is highly variable including: Capture IgM/IgG ratios greater than 1.78 [8], 1.2 [9] and 1.4 [10] to define primary infections; Capture IgG/IgM ratios greater than 1.10 [11] and 1.14 [12] to define secondary infections; NS1 specific IgG titres [13], absolute IgG titres [14] and/or measures of IgG avidity [15–17]; and Indirect IgG ELISA results [18]. In most of these reported studies only small numbers of participants were involved and the data used to define the outcome (primary versus secondary disease), and to develop the corresponding diagnostic algorithm, relied on serological tests performed on the same samples; while the specific tests were usually different, some degree of linkage is inevitable given the coordinated nature of immune responses to infection within individuals. In addition the available algorithms have only occasionally accounted for the known kinetics of antibody responses during acute infection [19, 20]. A combined approach has sometimes been adopted, in one study a case was defined as primary if the IgM/IgG ratio was above 1.78, secondary if the ratio was under 1.2 and indeterminate if it fell between these values [21].

Recently, the various laboratory methods used to diagnose dengue were evaluated to define the best approach within specified time-periods during infection [22, 23]. The aim of this study was to characterise the influence of illness day on a variety of methods currently used to determine immune status in confirmed dengue cases. If applicable, we also wished to develop simple and practical models to differentiate primary from secondary dengue on specimens obtained at any time during the acute illness. To avoid the circularity mentioned above, we elected to use plaque reduction neutralisation tests (PRNTs) performed 6 months after the acute illness episode to define immune status.

## Methods

### Patients and samples

Laboratory confirmed dengue patients aged 5–25 years who had enrolled into one of several clinical studies carried out at the Hospital for Tropical Diseases (HTD) in Ho Chi Minh City [24, 25] were invited to participate in this study. This work, and all the associated research studies, were approved by the Ethical Committee at HTD and the Oxford Tropical Research Ethics Committee. The original studies had included both outpatient and inpatient recruitment and focused on early enrolment of children and young adults presenting with clinical syndromes consistent with dengue. In all studies, clinical information was recorded daily and a 1 ml research blood sample was obtained each day until defervescence, or, for hospitalized individuals, until discharge. Illness day was counted from 1 (Day1, Day2, Day3 etc.) where Day1 was the reported day of fever onset.

Study participants with confirmed dengue (positive dengue RT-PCR [26]) were contacted and invited to attend a follow-up visit approximately 6 months after their acute illness. Following written consent by the patient or their parent/guardian, a short questionnaire was completed, and individuals who had been well since the initial acute dengue study were asked to provide a 2 ml blood sample.

### ELISA tests

Daily plasma samples obtained during the acute illness were assayed using the Panbio anti-dengue Indirect IgG ELISA, as per the manufacturer’s instructions, as well as in-house anti-dengue IgG and IgM capture ELISAs, as described elsewhere [24, 27]. For the in-house assays, the negative control was a pool of plasma from dengue naïve individuals, and the positive control was a pool of plasma from acute dengue patients. The cut-off for positivity was defined as 5 times the negative control value. The index unit was calculated as 10 times the ratio of the normalized value and the cut-off [24].

### PRNT assay and rule for differentiation of primary and secondary dengue

PRNT assays were conducted on the blood samples obtained 6 months after infection. PRNTs were performed at the Laboratory of Infectious Diseases, National Institute for Allergy and Infectious Disease (NIAID), National Institutes of Health (NIH), USA, as described elsewhere [28, 29]. PRNT_60_ titres (the reciprocal of the dilution at which the number of plaques was reduced by 60%) were used. A case was classified as primary when the PRNT_60_ titre to the infecting serotype (i.e. causing the recent infection) was > = 20 and the PRNT_60_ titre for all remaining serotypes was < 20. Cases were classified as secondary when the PRNT_60_ titre for the infecting serotype was > = 20 with the PRNT_60_ titre for at least one other serotype either > = 40 or > = the PRNT_60_ titre of the infecting serotype (whichever was lower). Other cases were classified as indeterminate.

### Statistical analysis

Cases with a clearly defined immune status according to PRNT were included for development of the algorithms. The outcome assessed was the ability to differentiate between primary and secondary infections, as defined by the convalescent PRNT titres, using only acute serology results. Models were developed for each marker separately - Panbio Indirect IgG, in-house capture IgG, in-house capture IgM, and in-house capture IgM/IgG ratio. The “all-inclusive” logistic models used the (log2 transformed) marker value and the corresponding illness day as predictors and included data from all available samples from Day2 to Day7 of illness. To account for within-patient correlation of measurements from the same patient on different illness days, a marginal logistic regression with independence working correlation was used. For each all-inclusive model, a cut-off-point was chosen as the value with the highest accuracy ([true positive + true negative]/total cases).

Potential interactions between marker levels and illness day, non-linearity of marker effects (modelled as flexible natural cubic spline functions with 4 degrees of freedom), or the effect of other covariates (sex, age, serotype, hospitalization and time of admission) were assessed using Wald-type tests. If adding an interaction term between marker level and illness day substantially improved model performance, it was added to the respective “all-inclusive model”.

The area under the ROC curve (AUC), accuracy, specificity, sensitivity, positive predictive value and negative predictive value (based on the cut-off that maximized accuracy) were used in assessing and comparing model performance. As the model complexity of the “all-inclusive” models was low compared to the amount of available data, we expected minimal over-fitting and therefore report apparent performance throughout. In addition, the all-inclusive models (including the cut-off selection) were validated using bootstrapping as described by Harrell [30], and temporal cross-validation. For temporal cross-validation, patients were divided into 5 equal groups by date of enrolment. The models were then developed on training sets including 4 of these patient groups, and validated on the remaining (5th) group of patients. The final performance was calculated as average performance of 5 validation assessments.

The performance of the all-inclusive models was also compared to various “time-specific” univariable and multivariable logistic regression models that utilised only marker values from Day3 (early-phase models), or Day6 (late-phase models), or both Day3 and Day6 (dual-phase models), and lastly all days from Day3 to Day6 together (Day3–6 models). In addition, several of the algorithms currently in use were assessed [10, 13].

All statistical analyses were performed in R version 3.1.1 (07/10/2014) [31]. Marginal logistic regression models were fitted with the R package geepack version 1.2–0 [32].

## Results

### Patient characteristics and associations with immune status

**Fig. 1:**
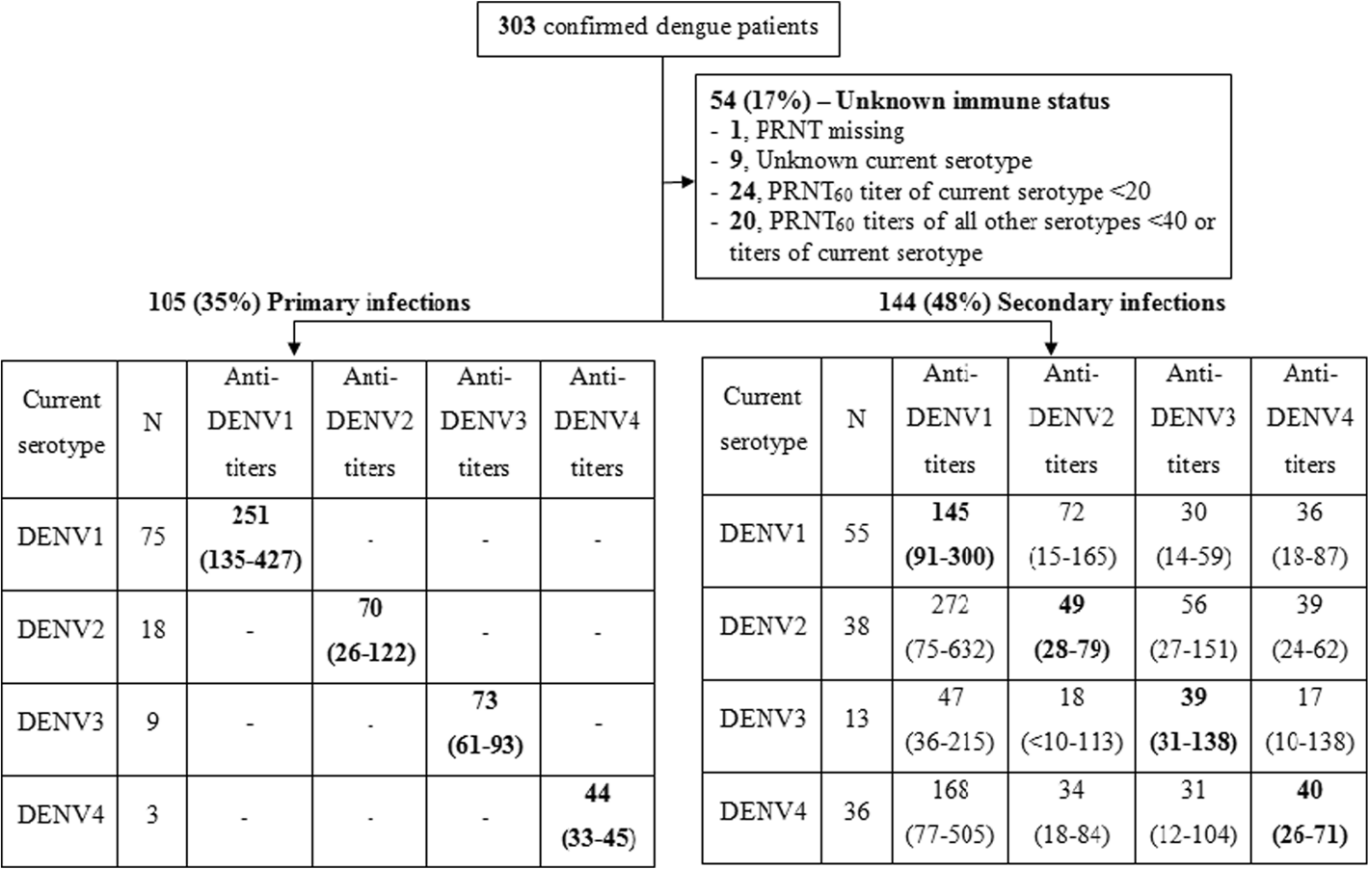
Summary of PRNT results and the classification of immune status. For each immune status category the PRNT titres are summarized in terms of median (IQR), with the current serotype shown in bold. In the blank cells titres were either unmeasurable or low (≥ 10 < 20)

Three hundred three confirmed dengue patients agreed to participate in this study. One patient was excluded because of missing PRNT titres, and 9 patients were excluded because the infecting serotype was not determined, leaving 293 patients included in the analysis. The median time to sampling for PRNT assay was 209 (IQR = 188–241) days from onset of fever. PRNT results by immune status and infecting serotype are summarised in Fig. 1; 105/293 (36%) and 144/293 (49%) patients were defined as having primary and secondary infections respectively. The remaining 44 patients (15%) could not be classified.

Demographic and virological information is presented in Table 1. Using univariate logistic regression, older age and female sex were more likely to be associated with secondary dengue (OR (95%CI) = 1.11(1.05–1.17) for each year of age and 2.79(1.61–4.83) for female patients, see Table 1). Male and female patients were not different in age. In addition, infections with DENV2 and DENV4 were more likely to be secondary than DENV1, with OR (95%CI) compared to DENV1 of 3.03(1.57–5.84) for DENV2 and 16.36(4.79–55.88) for DENV4. Illness day at presentation was not significantly associated with immune status (*p* = 0.1, logistic regression). Surprisingly, a higher proportion of secondary infections was seen among community (outpatient) participants compared to those hospitalised (67% versus 50%). However this finding likely reflects demographic differences in the different study populations: overall, patients in the community studies were older than patients in the hospital-based studies with a median (IQR) age of 15(12, 20) years compared to 12(10, 14) years.

**Table 1:** Patient characteristics. Continuous variables are presented as median (IQR) and categorical variables are presented as number and percentage

		Primary	Secondary	Unknown	*p* value	Odds Ratio (95% CI)
		*N* = 105	*N* = 144	*N* = 44		
		(36%)	(49%)	(15%)		
Age (years)		13 (10.0–16.0)	14 (11.0–19.0)	14 (12.0–19.8)	< 0.001	1.11 (1.05–1.17)
Sex (% male)		79 (75)	76 (53)	35 (79)	< 0.001	2.79 (1.61–4.83)
Day of fever at enrolment, Number (%)	1	6 (6)	5 (4)	4 (9)	0.1	
	2	51 (49)	54 (38)	19 (43)		
	3	45 (43)	82 (57)	21 (48)		1.40 (0.94, 2.10)
	4	2 (2)	3 (2)	0 (0)		
	5	1 (1)	0 (0)	0 (0)		
Serotype Number (%)	1	75 (71)	55 (38)	8 (18)	< 0.001	–
	2	18 (17)	40 (28)	17 (40)		3.03 (1.57–5.84)
	3	9 (9)	13 (9)	7 (15)		1.96 (0.79–4.91)
	4	3 (3)	36 (25)	12 (27)		16.36 (4.79–55.88)

*P*-values are for comparisons between primary and secondary infections, using univariate logistic regression

**Fig. 2:**
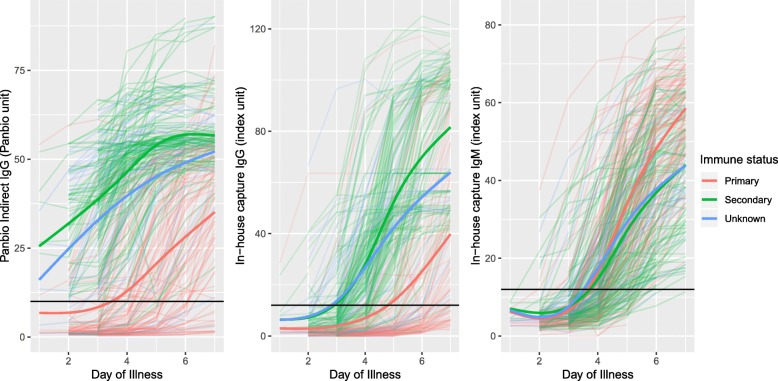
Antibody kinetics by immune status. Each thin line is an individual patient values, coloured by immune status group. The thick coloured line for each colour is the smoothed median for the relevant immune status group. The black horizontal lines indicate the cut-off for a positive result for each test

The kinetics of the antibody responses by illness day by immune status are shown in Fig. 2. IgG levels started to rise earlier and peaked at a higher level in secondary compared to primary infections, while IgM kinetics were quite similar.

### The all-inclusive models

For each of the different markers separately (results of IgG capture, IgM/IgG capture ratio and IgG indirect) the all-inclusive model used all the values for the relevant marker obtained from each of Day2 to Day7 (541 and 673 samples from 105 primary and 144 secondary infections, respectively). The all-inclusive models were developed using marginal regression, clustered by patient, with the following formula: Logit (secondary/primary) = Marker + DOI. By the Wald-type test, adding an interaction term between marker level and illness day, adjusting for other covariates, including serotype, or including the marker in a flexible, potentially non-linear way were all shown to have a significant effect in all of the all-inclusive models (all *p*-values< 0.001). However, the general performance of the various models changed very little, except for the model using the Panbio Indirect IgG with the interaction term. In this instance, the performance of the model with the interaction was better on Day6, accuracy of 0.77 and 0.84 for non-interaction and interaction models respectively (Table 4). Taking into account these effects, we chose three linear models for subsequent analysis. These models were models including marker and illness day for a) the in-house capture IgG and b) the in-house capture IgM/IgG ratio, and c) a model including the marker and the illness day and an interaction term between the marker and illness day for the Panbio Indirect IgG.

The “all-inclusive model” based on the Panbio Indirect IgG and including the interaction term gave the best performance compared to the other models by all metrics assessed (see Table 2). The Panbio Indirect IgG model had the best AUC of 0.90 (95%CI = 0.88–0.92) and accuracy of 0.85 compared to the models with the other markers (AUC of 0.86(0.83–0.89) and accuracy of 0.84 for in-house capture IgG, and AUC of 0.88(0.85–0.90) and accuracy of 0.84 for in-house capture IgM/IgG). As expected, the model based on in-house capture IgM values was not suitable for defining immune status with an AUC of only 0.55(0.51–0.59) and an accuracy of only 0.58.

**Table 2:** Performance of the all-inclusive models to discriminate primary from secondary dengue

Marker	AUC (95% CI)	Accuracy	Sens.	Spec.	PPV	NPV
Panbio Indirect IgG	0.90 (0.88–0.92)	0.85	0.91	0.78	0.84	0.86
In-house capture IgG	0.86 (0.83–0.89)	0.84	0.89	0.77	0.84	0.84
In-house capture IgM	0.55 (0.51–0.59)	0.58	0.81	0.28	0.60	0.53
In-house capture IgM/IgG ratio	0.88 (0.85–0.90)	0.84	0.90	0.77	0.83	0.85

For this and subsequent similar tables AUC area under ROC curve, *Sens* sensitivity; *Spec*. specificity, *PPV* positive predictive value, *NPV* negative predictive value. Cut-offs were selected to maximize accuracy as described in the statistical methods section

The cut-offs for each parameter on each day, as derived from the three best all-inclusive models are presented in Table 3. For the Panbio Indirect IgG, and the in-house capture IgG, an infection would be defined as secondary if the value equals or exceeds the cut-off for that day, while for the capture IgM/IgG ratio, an infection would be defined as primary if the ratio equals or exceeds the cut-off for that day. Classifying based on the cut-offs for positivity defined by the manufacturer for the indirect assay (i.e. primary classified as < 11 Panbio units), the classification would be the same as our classification on Day2, but from Day3 onwards an increasing percentage of cases (20% on Day3 up to 79% on Day7) classified as primary using our algorithm would be classified as secondary using just the manufacturer’s positivity cut-off.

**Table 3:** Cut-offs for the selected parameters, derived from the all-inclusive models on each individual day of illness

Day of illness	Panbio Indirect IgG (Panbio unit)	In-house Capture IgG (Index unit)	In-house Capture IgM/IgG ratio
2	9.3	1.6	1.8
3	19	3.1	1.6
4	28	6.1	1.4
5	37	12	1.3
6	46	24	1.1
7	53	47	1.0

We also took the cut-off points for each day from the all-inclusive models and evaluated all metrics (except AUC) separately by illness day (Table 4). Panbio Indirect IgG performance proved to be better in the early compared to the late phase, with decreasing accuracy from Day2 to Day7: 0.86 to 0.79. Conversely the capture IgG and capture IgM/IgG proved to be better in the late compared to the early phase (capture IgG accuracy: Day2 to Day7: 0.76 to 0.80 and capture IgM/IgG accuracy: Day2 to 7: 0.80 to 0.83).

**Table 4:** Performance of the all-inclusive models on each individual day of illness, from Day2 to Day7

	Performance				
	Accuracy	Sens.	Spec.	PPV	NPV
Day 2 (56 primary vs 55 secondary dengue)					
Panbio Indirect IgG	0.86	0.91	0.80	0.82	0.90
In-house capture IgG	0.76	0.87	0.64	0.71	0.84
In-house capture IgM/IgG ratio	0.80	0.78	0.82	0.81	0.79
Day 3 (102 primary vs 135 secondary dengue)					
Panbio Indirect IgG	0.86	0.86	0.86	0.89	0.82
In-house capture IgG	0.83	0.81	0.85	0.88	0.77
In-house capture IgM/IgG ratio	0.80	0.79	0.80	0.84	0.75
Day 4 (102 primary vs 136 secondary dengue)					
Panbio Indirect IgG	0.86	0.86	0.90	0.80	0.85
In-house capture IgG	0.85	0.89	0.80	0.86	0.85
In-house capture IgM/IgG ratio	0.82	0.87	0.77	0.83	0.81
Day 5 (101 primary vs 140 secondary dengue)					
Panbio Indirect IgG	0.87	0.94	0.77	0.85	0.90
In-house capture IgG	0.85	0.94	0.73	0.83	0.89
In-house capture IgM/IgG ratio	0.86	0.89	0.81	0.87	0.84
Day 6 (98 primary vs 116 secondary dengue)					
Panbio Indirect IgG	0.84	0.93	0.72	0.80	0.90
In-house capture IgG	0.81	0.94	0.66	0.77	0.90
In-house capture IgM/IgG ratio	0.85	0.91	0.79	0.83	0.88
Day 7 (82 primary vs 91 secondary dengue)					
Panbio Indirect IgG	0.79	0.74	0.84	0.84	0.74
In-house capture IgG	0.80	0.93	0.66	0.75	0.90
In-house capture IgM/IgG ratio	0.83	0.96	0.68	0.77	0.93

### Validation

For both the temporal and bootstrap validation methods, the performance of all metrics for all models was very similar to their performance during the development process. For example, there was an accuracy of 0.90 from the bootstrapping validation and 0.83 from the temporal validation, compared to 0.85 in the development process (Additional file 1: Table S1). This indicates that models were not over-optimized or over-fitted.

### Comparisons with time-specific models

We compared the performance of the all-inclusive models with the corresponding time-specific models: early-phase, late-phase, dual-phase and Day3–6 models. The performances of the all-inclusive models on Day3 and Day6 (Table 4) were comparable to the performances of the corresponding early-phase and late-phase models (Table 5). The performance of the dual-phase and Day3–6 models was a little better than the all-inclusive models (Table 4).

**Table 5:** Performance of early-phase, late-phase, dual-phase and Day3–6 models

	Performance					
	AUC (95% CI)	Accuracy	Sens.	Spec.	PPV	NPV
Early-phase models (102 primary vs 135 secondary dengue)						
Panbio Indirect IgG	0.89 (0.84–0.93)	0.87	0.91	0.82	0.87	0.88
In-house anti-E indirect IgG	0.81 (0.75–0.86)	0.77	0.73	0.82	0.84	0.69
In-house capture IgG	0.83 (0.77–0.89)	0.83	0.81	0.86	0.89	0.77
In-house capture IgM/IgG ratio	0.82 (0.76–0.88)	0.82	0.86	0.76	0.83	0.80
Late-phase models (98 primary vs 116 secondary dengue)						
Panbio Indirect IgG	0.89 (0.85–0.93)	0.85	0.93	0.74	0.81	0.90
In-house anti-E indirect IgG	0.73 (0.67–0.80)	0.71	0.82	0.59	0.70	0.73
In-house capture IgG	0.85 (0.80–0.91)	0.85	0.92	0.77	0.82	0.89
In-house capture IgM/IgG ratio	0.90 (0.86–0.94)	0.86	0.94	0.77	0.83	0.91
Dual-phase models (95 primary vs 109 secondary dengue)						
Panbio Indirect IgG	0.90 (0.86–0.94)	0.86	0.89	0.83	0.86	0.87
In-house anti-E indirect IgG	0.79 (0.72–0.85)	0.74	0.72	0.77	0.78	0.70
In-house capture IgG	0.86 (0.81–0.92)	0.85	0.91	0.79	0.83	0.88
In-house capture IgM/IgG ratio	0.90 (0.86–0.94)	0.86	0.93	0.79	0.83	0.90
Days 3–6 models (90 primary vs 102 secondary dengue)						
Panbio Indirect IgG	0.92 (0.88–0.96)	0.88	0.92	0.82	0.85	0.90
In-house anti-E indirect IgG	0.82 (0.76–0.89)	0.79	0.79	0.79	0.81	0.77
In-house capture IgG	0.89 (0.84–0.94)	0.88	0.91	0.83	0.86	0.89
In-house capture IgM/IgG ratio	0.93 (0.89–0.96)	0.88	0.95	0.79	0.84	0.93

### Comparisons with selected existing algorithms

The established algorithms of Innis and Shu using the IgM/IgG ratio showed better performance in the late phase (accuracy on Day6 of 0.82 (Innis) and 0.84 (Shu)) compared to the early phase (accuracy on Day3 of 0.76 (Innis) and 0.74 (Shu)). The combined algorithm, i.e. the algorithm using both cut-offs of 1.2 and 1.78, gave good stable performance in both early and late phases (accuracy of 0.80 and 0.82 on Day3 and Day6 respectively). However 6% of cases could not be classified with this strategy.

## Discussion

Differentiating between primary and secondary dengue infections is important especially in pathogenesis research and for epidemiological surveillance, but also potentially in clinical practice. In this study, we evaluated the time-course of different sero-diagnostic responses during acute dengue in 1214 daily specimens from 249 patients, and developed various models to differentiate between primary and secondary infections, using the PRNT responses 6 months after infection as gold standard. For all assays, the best fitting models estimated a different cut-off value for different days of illness, confirming how rapidly the immune response changes during acute infection.

The all-inclusive models using the Panbio Indirect IgG, in-house capture IgG, and in-house capture IgM/IgG ratio performed well, both in general over the illness course, and when derived on any day from Day2 to Day7 (accuracy of 0.8–0.85 in differentiating between primary and secondary dengue). Although the dual-phase and Day3–6 models were a little better, the cost and practical difficulties associated with additional sampling and testing, limit the relevance of this approach. We also assessed the performance of the two most widely used of the established algorithms [10, 13] using our dataset. Innis and Shu’s algorithms showed better performance in the late phase, which may reflect the time point of sample collection in the studies used to define these algorithms. The combined strategy (which has never been formally assessed), using both Innis’ and Shu’s cut-offs of 1.78 and 1.2, gave comparable performance to the all-inclusive model based on the in-house capture IgM/IgG ratio. This is consistent with the findings for the cut-offs derived from the in-house capture IgM/IgG all-inclusive model, which ranged from 1.8 on Day2 (similar to Innis) to 1.0 on Day7. Using the combined algorithm, however, immune status cannot be defined for patients where the IgM/IgG ratio falls between 1.2 and 1.78, an issue that is circumvented with the models developed here. The 1.4 cut-off used by Kuno is the same as the value we estimate on Day4, and the 1.2 used by Shu falls between our estimates from Day5 and Day6. Therefore application of Innis’ algorithm overestimates secondary infections from Day2 onwards, while both Kuno and Shu algorithms overestimate primary infections early in illness, and overestimate secondary infections later in illness. Although here we assessed our in-house capture assays, the same principles are likely to apply to commercial capture ELISA assays, particularly when considering the IgM/IgG ratio.

It is important to note however, that these models were developed using confirmed dengue cases, and further work will be needed to assess their utility when dengue is suspected but not yet confirmed and where Zika and chikungunya may be circulating. Although rapid NS1 testing is available in some clinical settings, there is usually a delay for RT-PCR confirmation. IgM and IgG responses as measured by capture ELISA did not rise above the threshold for a positive response until after Day4 in most cases (Fig. 2), and the reliability of measurements that fall below the positive threshold in any assay is questionable [10]. By contrast indirect ELISA methodology measures much lower concentrations of dengue specific IgG, which should already be present in the early days of an infection in individuals previously exposed to DENV, but not in naïve individuals. In line with this, the all-inclusive model based on Panbio Indirect IgG without the interaction with illness day showed very good performance in the early phase, but less good performance later in illness course; however the interaction term helped to improve performance at this later time point. At present, the Panbio Indirect IgG ELISA is mainly used in a qualitative way (with a positive or negative outcome) and this assessment extends the utility of the test in classifying primary and secondary infections in confirmed dengue cases.

Solely based on measures of goodness of fit, a model based on the Panbio Indirect IgG would be appropriate for early specimens (illness day≤4), while a model based on capture IgG or IgM/IgG ratios would be more suitable for late acute specimens (illness day> 4). In practice, the choice will likely depend on the setting. In intervention trials or cohort studies, where subjects are being closely monitored, individuals are more likely to be tested early in infection, so the Panbio Indirect IgG model may be preferable. However, in tertiary hospital settings, patients tend to present later in illness course, so the capture IgG or IgM/IgG ratio model is likely to be preferable. Whichever test is used, our results show that the incorporation of day of illness into the algorithm means that determining primary or secondary status can be done using a single sample on each day of acute infection.

Several potential limitations to this study need to be considered. First, we developed a large number of models on the same dataset. However, this is the most extensive assessment of its kind to date involving 249 individuals, and we planned all statistical analyses in advance. In addition we included validation using bootstrapping and temporal cross-validation to show that the reported models did not over-fit the data or lead to over-optimistic performance claims. Second, we chose to use PRNTs 6 months after infection as the gold standard for developing the models, not HI during infection as has been used conventionally in previous studies. One reason for this choice was to try to avoid circularity – i.e. use of serological data collected at the same time-point both for developing the diagnostic algorithms and for assessing outcome. In addition, although cross-reactivity is recognized as a potential issue for all serological tests for dengue, neutralization assays are considered to have the greatest specificity to differentiate between flaviviruses. Japanese Encephalitis virus (JEV) transmission occurs in Vietnam, with increasing uptake of vaccination in recent years. However since the PRNTs were dengue specific, cross-reactivity should be reduced, and there were still many individuals who were indirect IgG negative at the time of the acute illness episode, suggesting that widespread JE vaccination has not yet had a major impact. DENV neutralizing antibody level is also not thought to be influenced by prior flavivirus vaccination (yellow fever or Japanese encephalitis virus) if performed in late convalescence [33, 34].

With the recent widespread transmission of Zika virus globally, we must also consider the possibility of Zika cross-reactivity in both the acute samples and in the PRNTs. Though we know little about Zika circulation in Vietnam at the time this study was undertaken, we cannot exclude the possibility that those classified as a secondary dengue infection had instead experienced a first Zika infection. Overall however, we identified more secondary cases compared to primary, consistent with the theory that the outcome of a true secondary dengue exposure will be more severe compared to a first. However, more research is needed to understand how exposure to other flaviviruses changes the results of dengue serology and infection outcome.

Another limitation of using the PRNT assay is that the differences in titres to each serotype (generally higher titres against DENV1 compared to other serotypes) meant that more DENV2, DENV3 and DENV4 infections were classified as indeterminate immune status and therefore excluded from the analysis. Although this might bias the performance of the all-inclusive models, when we applied these models to specimens obtained during the acute illness from this indeterminate group of 54 patients, the majority (70–74%) were classified as secondary infections, in agreement with visual inspection of marker dynamics shown in Fig. 2. In addition, when using the models to define immune status for all 303 patients included in the study, 59–63% were classified as secondary dengue, a very similar proportion to the 144/249 (59%) classified as secondary dengue based only on the 6 month PRNT data. This suggests that the indeterminate group were similar to the whole study group.

## Conclusions

In summary, in this study we developed diagnostic algorithms aimed at differentiating between primary and secondary dengue infections during the acute phase of illness. By describing in fine detail acute serological responses in this large group of confirmed dengue patients we have contributed to the overall knowledge of the typical patterns seen, and confirmed the rapid evolution of responses during the first week of illness. We tested models based on a variety of assays and found that models using our in-house capture IgG, the capture IgM/IgG ratio or the Panbio Indirect IgG all performed well across sequential days of illness. We illustrate that primary vs secondary discrimination on single samples from different days of illness can be improved by taking the change in titres over time into account. The findings suggest that the all-inclusive model based on the commercial Panbio Indirect IgG kit may be suitable for defining immune status in the early phase of illness, while the capture IgG or IgM/IgG models are preferable after Day4 of illness. However, more importantly the work emphasizes the variability and uncertainty surrounding use of the current algorithms. The participants in our studies were mainly children and young adults reflecting the epidemiology of symptomatic dengue in southern Vietnam; the underlying principles should be similar in other populations with different dengue endemicity, but local evaluation and adaptation of the models/cutoffs would likely be necessary before application of these models in other settings.

## Additional file

Additional file 1: **Table S1.** Validation of the all-inclusive models. (PDF 87 kb)

## Abbreviations

AUC: Area under the ROC curve
DENV: Dengue virus
ELISA: Enzyme-linked immunosorbent assay
HI: Haemagglutination Inhibition
HTD: Hospital for Tropical Diseases
NPV: Negative predictive value
PPV: Positive predictive value
PRNT: Plaque reduction neutralisation tests
ROC: Receiver operating characteristic

## References

[1] Bhatt S, Gething PW, Brady OJ, Messina JP, Farlow AW, Moyes CL, et al. The global distribution and burden of dengue. Nature. 2013 [cited 2014 Jan 20];496:504–7. Available from: https://www.ncbi.nlm.nih.gov/pmc/articles/PMC3651993/10.1038/nature12060 PMC3651993 23563266

[2] Thein S, Aung MM, Shwe TN, Aye M, Zaw A, Aye K (1997). Risk factors in dengue shock syndrome. Am J Trop Med Hyg.

[3] Endy TP, Nisalak A, Chunsuttitwat S, Vaughn DW, Green S, Ennis FA (2004). Relationship of preexisting dengue virus (DV) neutralizing antibody levels to viremia and severity of disease in a prospective cohort study of DV infection in Thailand. J Infect Dis.

[4] Priyadarshini D, Gadia RR, Tripathy A, Gurukumar KR, Bhagat A, Patwardhan S, Sandberg JK (2010). Clinical Findings and Pro-Inflammatory Cytokines in Dengue Patients in Western India: A Facility-Based Study.

[5] Tricou V, Minh NN, Farrar J, Tran HT, Simmons CP (2011). Kinetics of viremia and NS1 antigenemia are shaped by immune status and virus serotype in adults with dengue. PLoS Negl Trop Dis.

[6] Rothman AL (2009). T Lymphocyte Responses to Heterologous Secondary Dengue Virus Infections. Ann N Y Acad Sci.

[7] Vaughn DW, Green S, Kalayanarooj S, Innis BL, Nimmannitya S, Suntayakorn S, et al. Dengue viremia titer, antibody response pattern, and virus serotype correlate with disease severity. J Infect Dis. 2000:181, 2–9. [cited 2016 Jun 23]. Available from: http://www.ncbi.nlm.nih.gov/pubmed/10608744. 10.1086/315215 10608744

[8] Innis BL, Nisalak A, Nimmannitya S, Kusalerdchariya S, Chongswasdi V, Suntayakorn S (1989). An enzyme-linked immunosorbent assay to characterize dengue infections where dengue and Japanese encephalitis co-circulate. Am J Trop Med Hyg.

[9] Shu P-Y, Chen L-K, Chang S-F, Yueh Y-Y, Chow L, Chien L-J, et al. Potential application of nonstructural protein NS1 serotype-specific immunoglobulin G enzyme-linked immunosorbent assay in the seroepidemiologic study of dengue virus infection: correlation of results with those of the plaque reduction neutralization test. J Clin Microbiol. 2002;40:1840–4. [cited 2014 Feb 14]. Available from: https://www.ncbi.nlm.nih.gov/pubmed/11980973 10.1128/JCM.40.5.1840-1844.2002 PMC130672 11980973

[10] Kuno G, Gómez I, Gubler DJ (1991). An ELISA procedure for the diagnosis of dengue infections. J Virol Methods.

[11] Changal KH, Raina AH, Raina A, Raina M, Bashir R, Latief M, et al. Differentiating secondary from primary dengue using IgG to IgM ratio in early dengue: an observational hospital based clinico-serological study from North India. BMC Infect Dis BioMed Central. 2016;16:715. [cited 2016 Dec 12] Available from: https://www.ncbi.nlm.nih.gov/pmc/articles/PMC5127094/. 10.1186/s12879-016-2053-6. 10.1186/s12879-016-2053-6 PMC5127094 27894268

[12] Cucunawangsih, NPH L, Kurniawan A. Immunoglobulin G (IgG) to IgM ratio in secondary adult dengue infection using samples from early days of symptoms onset. BMC Infect Dis. 2015;15:–276. [cited 2017 Dec 8]. Available from: http://www.ncbi.nlm.nih.gov/pubmed/26193930 10.1186/s12879-015-1022-9 PMC4509644 26193930

[13] Shu P-Y, Chen L-K, Chang S-F, Yueh Y-Y, Chow L, Chien L-J, et al. Comparison of capture immunoglobulin M (IgM) and IgG enzyme-linked immunosorbent assay (ELISA) and nonstructural protein NS1 serotype-specific IgG ELISA for differentiation of primary and secondary dengue virus infections. Clin Diagn Lab Immunol. 2003; [cited 2016 Dec 12];10:622–30. Available from: http://www.ncbi.nlm.nih.gov/pubmed/12853395 10.1128/CDLI.10.4.622-630.2003 PMC164246 12853395

[14] Vaughn DW, Nisalak A, Solomon T, Kalayanarooj S, Nguyen MD, Kneen R (1999). Rapid serologic diagnosis of dengue virus infection using a commercial capture ELISA that distinguishes primary and secondary infections. Am J Trop Med Hyg.

[15] Matheus S, Deparis X, Labeau B, Lelarge J, Morvan J, Dussart P (2005). Discrimination between Primary and Secondary Dengue Virus Infection by an Immunoglobulin G Avidity Test Using a Single Acute-Phase Serum Sample. J Clin Microbiol.

[16] de Souza VAUF, Fernandes S, Araújo ES, Tateno AF, Oliveira OMNPF, Oliveira RR (2004). Use of an immunoglobulin G avidity test to discriminate between primary and secondary dengue virus infections. J Clin Microbiol.

[17] Prince HE, Matud JL, Lieberman JM. Dengue virus immunoglobulin M detection in a reference laboratory setting during the 2010 dengue virus outbreak on Caribbean islands. Clin Vaccine Immunol. 2011;18:1104–7. [cited 2014 Feb 14]. Available from: https://www.ncbi.nlm.nih.gov/pmc/articles/PMC3147312/10.1128/CVI.05096-11 PMC3147312 21613462

[18] Inoue S, Alonzo MTG, Kurosawa Y, Mapua CA, Reyes JD, Dimaano EM (2010). Evaluation of a Dengue IgG Indirect Enzyme-Linked Immunosorbent Assay and a Japanese Encephalitis IgG Indirect Enzyme-Linked Immunosorbent Assay for Diagnosis of Secondary Dengue Virus Infection. Vector-Borne Zoonotic Dis.

[19] Cordeiro MT, Braga-Neto U, Nogueira RMR, Marques ETA, Gubler D, Rigau-Perez J, et al. Reliable Classifier to Differentiate Primary and Secondary Acute Dengue Infection Based on IgG ELISA. Ng LFP, editor. PLoS One. Public Library of Science; 2009;4:e4945 [cited 2016 Dec 12]. Available from: DOI:10.1371/journal.pone.0004945 10.1371/journal.pone.0004945 PMC2660412 19340301

[20] Falconar AKI, de Plata E, CME R-V (2006). Altered Enzyme-Linked Immunosorbent Assay Immunoglobulin M (IgM)/IgG Optical Density Ratios Can Correctly Classify All Primary or Secondary Dengue Virus Infections 1 Day after the Onset of Symptoms, when All of the Viruses Can Be Isolated. Clin Vaccine Immunol.

[21] Fox A, Le NMH, Simmons CP, Wolbers M, Wertheim HFL, Pham TK, et al. Immunological and viral determinants of dengue severity in hospitalized adults in Ha Noi, Viet Nam. PLoS Negl Trop Dis. 2011;5:e967. [cited 2014 Feb 6]. Available from: https://www.ncbi.nlm.nih.gov/pmc/articles/PMC3046970/. 10.1371/journal.pntd.0000967 PMC3046970 21390156

[22] Hunsperger EA, Muñoz-Jordán J, Beltran M, Colón C, Carrión J, Vazquez J (2016). Performance of Dengue Diagnostic Tests in a Single-Specimen Diagnostic Algorithm. J Infect Dis.

[23] Peeling RW, Olliaro P (2016). Reimagining the Future of the Diagnosis of Viral Infections. J Infect Dis.

[24] Duyen HTL, Ngoc TV, Ha DT, Hang VTT, Kieu NTT, Young PR, et al. Kinetics of plasma viremia and soluble nonstructural protein 1 concentrations in dengue: differential effects according to serotype and immune status. J Infect Dis. 2011;203:1292–300. [cited 2016 Apr 19]. Available from: https://www.ncbi.nlm.nih.gov/pmc/articles/PMC3069728/10.1093/infdis/jir014 PMC3069728 21335562

[25] Tam DTH, Ngoc TV, Tien NTH, Kieu NTT, Thuy TTT, Thanh LTC, et al. Effects of short-course oral corticosteroid therapy in early dengue infection in Vietnamese patients: a randomized, placebo-controlled trial. Clin Infect Dis. 2012;55:1216–24. [cited 2016 Apr 19]. Available from: https://www.ncbi.nlm.nih.gov/pmc/articles/PMC3466094/10.1093/cid/cis655 PMC3466094 22865871

[26] Simmons CP, Popper S, Dolocek C, Chau TNB, Griffiths M, Dung NTP, et al. Patterns of host genome-wide gene transcript abundance in the peripheral blood of patients with acute dengue hemorrhagic fever. J Infect Dis. 2007;195:1097–107 [cited 2017 Feb 14]. Available from: https://www.ncbi.nlm.nih.gov/pubmed/17357045. 10.1086/512162. 10.1086/512162 PMC4042601 17357045

[27] Cardosa MJ, Wang SM, Sum MSH, Tio PH (2002). Antibodies against prM protein distinguish between previous infection with dengue and Japanese encephalitis viruses. BMC Microbiol.

[28] Chau TN, Hieu NT, Anders KL, Wolbers M, Lien le B, Hieu LT (2009). Dengue virus infections and maternal antibody decay in a prospective birth cohort study of Vietnamese infants. J Infect Dis.

[29] Durbin AP, McArthur J, Marron JA, Blaney JE, Thumar B, Wanionek K, et al. The live attenuated dengue serotype 1 vaccine rDEN1Delta30 is safe and highly immunogenic in healthy adult volunteers. Hum Vaccin 2:167–73[cited 2014 Feb 13]. Available from: http://www.ncbi.nlm.nih.gov/pubmed/17012875. 10.4161/hv.2.4.2944 17012875

[30] Harrell F. Regression modeling strategies: with applications to linear model, Logistic Regression and Survival Analysis. New York: Springer; 2001.

[31] Team R. R Development Core Team. R A Lang Environ Stat Comput. 2013;55:275–86. Available from: https://www.r-project.org/

[32] Halekoh U, Højsgaard S, Yan J. The R Package geepack for Generalized Estimating Equations. JSS J Stat Softw. 2006;15 [cited 2016 Dec 12]. Available from: https://www.jstatsoft.org/article/view/v015i02

[33] Maeda A, Maeda J (2013). Review of diagnostic plaque reduction neutralization tests for flavivirus infection. Vet J.

[34] Mansfield KL, Horton DL, Johnson N, Li L, Barrett ADT, Smith DJ (2011). Flavivirus-induced antibody cross-reactivity. J Gen Virol.

