# Supplementary material for: Methods to discriminate primary from secondary dengue during acute symptomatic infection
Source: BMC Infect Dis. 2018 Aug 7;18:375. doi: 10.1186/s12879-018-3274-7 (PMC6081805; doi:10.1186/s12879-018-3274-7)
Supplement: Supplementary file 1 — Table S1. Validation of the all-inclusive models. (PDF 87 kb) [file 12879_2018_3274_MOESM1_ESM.pdf]

Supplementary Information

Table S1: Validation of the all-inclusive models

	**Performance**				
	**Accuracy**	**Sens.**	**Spec.**	**PPV**	**NPV**
**Bootstrapping validation**					
Panbio Indirect IgG	0.90	0.85	0.93	0.75	0.83
In-house capture IgG	0.86	0.84	0.87	0.80	0.85
In-house capture IgM/IgG ratio	0.88	0.84	0.90	0.77	0.84
**Temporal validation**					
Panbio Indirect IgG	0.83	0.88	0.77	0.82	0.85
In-house capture IgG	0.80	0.86	0.75	0.80	0.80
In-house capture IgM/IgG ratio	0.82	0.86	0.78	0.82	0.81
